# Caries Experience and Erosive Tooth Wear in Finnish Men Conscripts 2021: A Cross-Sectional Study

**DOI:** 10.3390/dj10070122

**Published:** 2022-07-02

**Authors:** Pertti Patinen, Tarja Tanner, Mika Huttunen, Annakaisa Muhonen, Sari Räsänen, Pernelle Moilanen, Jari Päkkilä, Vuokko Anttonen, Antti Kämppi

**Affiliations:** 1 Research Unit of Oral Health Sciences, University of Oulu, Aapistie 5, 90220 Oulu, Finland; pertti.patinen@fimnet.fi (P.P.); tarja.tanner@oulu.fi (T.T.); mika.j.huttunen@mil.fi (M.H.); pernelle_moilanen@hotmail.com (P.M.); vuokko.anttonen@oulu.fi (V.A.); 2 Finnish Defence Forces, Centre for Military Medicine, Tykkikentäntie 1, 11311 Riihimäki, Finland; annakaisa.muhonen@mil.fi (A.M.); sari.m.rasanen@mil.fi (S.R.); 3 Research Unit of Mathematical Sciences, University of Oulu, Pentti Kaiteran Katu 1, 90014 Oulu, Finland; jari.pakkila@oulu.fi; 4 Department of Oral and Maxillofacial Diseases, University of Helsinki, Haartmaninkatu 1, 00014 Helsinki, Finland

**Keywords:** caries burden, conscripts, erosive tooth wear

## Abstract

Background: In Finland, the development of oral health in young, 19- to 21-year-old males regarding restorative treatment need seems to have slowed down according to cross-sectional conscript studies between 1976 and 2011. At the individual level, the mean number of decayed teeth (DT > 0) has also steadily continued to decline. In Finland, military service is mandatory, and around 85% of males complete it. The aim of this cross-sectional study was to investigate the oral health status of young men at the beginning of the 2020s. Methods: The data were collected in July 2021 in the eight biggest Finnish Defence force garrisons by ten calibrated dentists serving in the ranks. The inclusion criteria for this study were a year of birth between 2000 and 2002 and male gender (n = 508). Third molars were included. Restorative treatment need was evaluated using ICDAS scoring. The study was designed according to the STROBE guidelines. Results: Mean DT value was 1.13 when third molars were included and 1.03 when they were excluded. Mean DMFT value was 3.23 and 2.98, respectively. The proportion of conscripts with DT > 0 was 36.4% and 34.8%, respectively. The prevalence of caries was concentrated among a small number of conscripts. Most (76.6%) had BEWE (basic erosive wear examination) of 0–2. Conscripts in the moderate and severe ETW (erosive tooth wear) groups (BEWE 3–13) comprised 23.5% of the cohort. None of the conscripts fell into the most severe group (BEWE 14–18). Conclusions: The oral health of conscripts has improved over the last ten years, and restorative treatment need has decreased significantly. Compared to previous studies, restorative treatment need was concentrated on an even smaller proportion of conscripts.

## 1. Introduction

Dental caries is one of the most common diseases worldwide and the most prevalent non-communicable disease of people [1,2,3]. Dental caries develops when bacteria in the oral cavity metabolize dietary sugars, producing acids. Acids demineralize the hard tissues of the teeth [4]. Manifestation of the disease as lesions can be prevented, and systemic efforts have been made towards oral health since the early 1970s in Finland. A positive oral health development among young men for restorative treatment need has been observed in cross-sectional studies between 1979 and 2011 [5,6,7,8,9] even if the progress seems to have slowed down [9]. The proportion of young men with decayed teeth (DT > 0) has also steadily continued to decline (Table 1). In contrast, Kassebaum et al. suggested that Finland belongs to weakest class considering age-standardized, untreated deciduous teeth caries [10]. Simultaneously, while the prevalence of caries and the restorative treatment need has decreased over the decades, the caries burden has become concentrated among an even smaller proportion of the population.

Restoring caries lesions and replacing restorations comprise the heaviest workload of dentists in Finland [11]. In addition to caries, tooth wear has been recognized as a major problem and a serious risk in maintaining good dental health in the long term. Tooth wear, most commonly caused by dental erosion (erosive tooth wear ETW), seems to be increasing in the younger population [12,13]. It has been suggested that 45% of the permanent teeth of young people [14] and up to 80% of the permanent teeth of adults would have erosive changes [13]. The increased prevalence of ETW in industrialized countries is thought to be due to consumption of acidic beverages or fresh fruits [13,15]. On the other hand, fruit juice consumption in Europe and Finland is decreasing [16,17]. In Finland, consumption of fresh fruits (citrus fruits excluded) [18] and soft drinks has increased 27.1% over the last ten years [19]. The prevalence of erosive tooth wear among Finnish adolescents or young adults has, to our knowledge, not been studied before. However, ETW was investigated among a Finnish cohort study group born in 1966 between 2012 and 2013. This study showed that the prevalence of erosive tooth wear is considerable: almost half of the middle-aged cohort members were found to need at least preventative care against the further progression of an erosive condition [20].

General conscription [21] in Finland is unique worldwide. Every male Finnish citizen is required to perform military or non-military (civil) service between the ages of 18 and 30. Military service is voluntary for women. During 2021, 21,770 men and 1173 women started their military service. These men represent roughly 76% of the national male age cohort. Around 2500 men undertake civil service annually [22]. In 2021, 4104 conscripts interrupted military service or switched to civil service [22]. The interruption is for medical reasons, in most cases [23]. Military service offers an exceptionally good opportunity to conduct a cross-sectional study examining the health of an age cohort. The compulsory medical examination at the beginning of conscription enables the collection of a very large quantity of data in a short time. This also provides a unique opportunity, both nationally and internationally, to conduct a high-quality cross-sectional study of the oral health of young adult men and the factors that correlate to their oral health.

Military service is the last time young men are gathered irrespective of their geographic location, socioeconomic status (SES), and educational background, allowing noteworthy cross-sectional studies to be conducted. There have been no new cross-sectional studies for this age and gender group since 2011.

The purpose of this cross-sectional study is to update the knowledge of restorative treatment need caused by dental caries and the prevalence of ETW among healthy Finnish young men in their twenties.

It is hypothesized that the number of conscripts needing restorative treatment has declined compared to the previous Finnish epidemiological study in this age group. The mean DT value per individual is also slightly reduced, and the majority of the caries burden is concentrated in an even smaller proportion of subjects. It is also hypothesized that erosive tooth wear is becoming more common among young men.

## 2. Materials and Methods

This cross-sectional study uses data acquired from the dental archives of the Finnish Defence Forces in July 2021. All potential male conscripts were randomly recruited to an oral examination as part of an obligatory general health examination. The COVID-19 restriction guidelines of the Finnish Ministry of Social Affairs and Health were followed.

The data were collected in the eight biggest garrisons of the Finnish Defence forces by ten calibrated dentists. Inclusion criteria were year of birth from 2000–2002, male gender, and participation consent. The total number of conscripts participating in the study was 508. The study population should be considered as a convenience sample because every conscript meeting the inclusion criteria received an oral examination.

During the first two weeks of military service, conscripts undergo a general health examination. COVID-19 restrictions meant that these examinations were held at the garrison service units or in the garrison hospital. From all those who were examined medically in a garrison hospital, at least one in five were picked from the alphabetically organized list and were invited to the oral examination. The conscripts selected were told about the oral health examination, and at this point, they could genuinely refuse or agree to participate. No refusals were met. After agreeing to participate in the study, the conscript filled in an acceptance form and gave their written consent.

The oral examination was conducted in a garrison dental care unit using the following instruments: unit light, oral mirror, probe, and gingival probe, according to WHO criteria for Oral Health Surveys [24]. Caries lesions were recorded according to the ICDAS criteria [25]. Lesions with ICDAS score 4–6 and active 3 were considered as needing restorative treatment (DT > 0). In uncertain cases, the dentists were advised to choose the more severe option. Filled tooth (FT) and missing tooth (MT) were recorded according to definitions of WHO [24]. Erosive tooth wear was recorded by using BEWE scoring. All surfaces of the teeth were examined, and the highest score in each sextant was recorded. BEWE sum scores were calculated by summarizing BEWE scores from each sextant: BEWE score 0 = no erosive tooth wear; score 1 = initial loss of surface texture; score 2 = distinct defect, hard tissue loss less than 50% of the surface area; score 3 = distinct defect, hard tissue loss at least 50% of the surface area [26]. Third molars were also included in the examination.

COVID-19 restrictions imposed very strict hygiene protocols, which were followed during the patient exchanges. As a result, the oral examination took approximately 15 min, which is considerably longer than in the previous similar study [27].

To ensure consistency between the dentists, a training session was held prior to the field study. Researchers went through a training session at which the study protocol, caries lesion diagnostic criteria (ICDAS), gum disease, and identification of tooth erosions were defined (BEWE). After the training session, the researchers performed a caries lesion identification test with 55 images of decayed teeth. Teeth were to be classified according to the ICDAS classification (severity/score and activity) [25]. The inter-examiner agreement and the intra-examiner agreement were calculated from the answers given. The inter-examiner agreement was 0.66 (range 0.38, 0.76), and the intra-examiner agreement was 0.76 (range 0.59, 0.90), respectively. The mean DT, MT, and FT values as well as DMFT values and standard deviation were calculated with and without wisdom teeth for conscripts. A Lorentz curve was drawn to illustrate polarization of dental caries burden among conscripts and Gini coefficient were calculated to illustrate deviation from perfect equality. The BEWE index was categorized into five risk groups (healthy 0, mild 1–2, moderate 3–8, severe 9–13, and very severe 14–18). Analyses were performed using SPSS version 27.0 (SPSS Inc., Chicago, IL, USA) and Office 365 Excel software 2108 (Microsoft Inc., Redmond, WA, USA).

## 3. Results

Among men aged 19–21 years who completed military service in 2021, the mean DT value was 1.13 (SD = 2.31) when wisdom teeth were included and 1.03 (SD = 2.17) when they were excluded. Respectively, mean DMFT values were 3.23 (SD = 3.83) and 2.98 (SD = 3.66) (Table 2).

**Figure 1:**
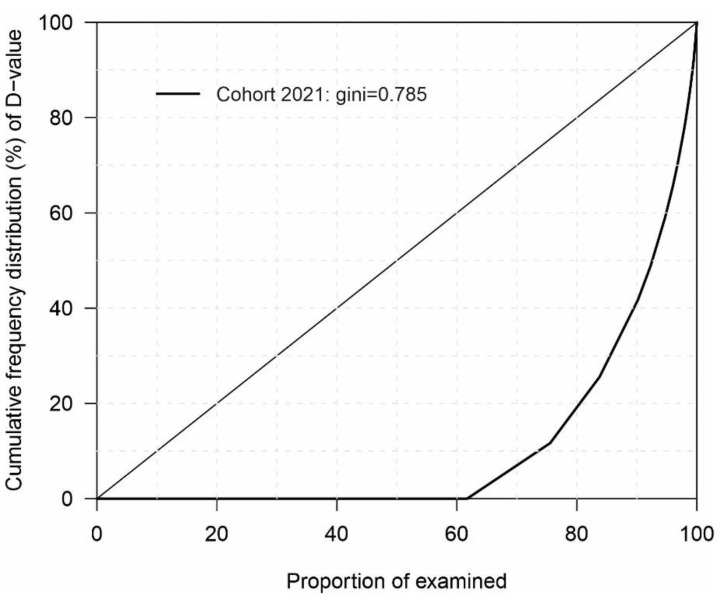
Polarization and concentration of caries burden among Finnish conscripts (Lorentz curve) 2021.

The proportion of conscripts with DT > 0 was 36.4% and 34.8% when third molars were included and excluded, respectively (Table 3). The prevalence of caries was concentrated among a small number of conscripts, with 23.6% of conscripts comprising 88.7% of the caries burden (Figure 1).

Large proportion of conscripts examined, 38.1%, had the first signs of erosive tooth wear (BEWE 1–2) visible in their dentition. Conscripts with moderate or severe ETW comprised 23.5% of all conscripts. None of the conscripts fell into the group of very severe ETW (BEWE = 14–18) (Table 4).

## 4. Discussion

**Figure 2:**
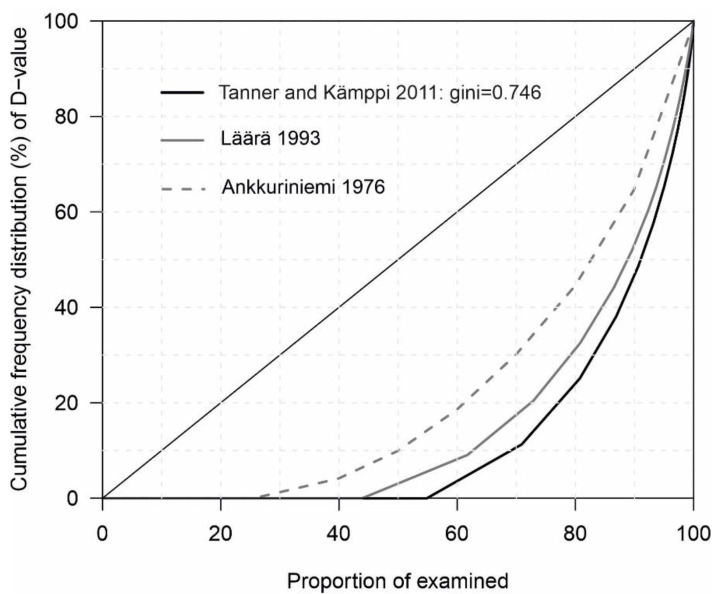
Summary of previous cross-sectional studies [5,6,7,8] polarizations (Lorentz curve) of Finnish conscripts. The curves of Ankkuriniemi and Läärä were manually adjusted from their dissertations.

The caries experience (D) has declined further in the group of males in their twenties compared to the study conducted in the last decade [7,8]. The same is true for mean DMFT values. Furthermore, the age-standardized prevalence of caries in Finland in permanent teeth has been found to be below or near the international average [28]. In the same study, the age-standardized prevalence of caries in deciduous teeth is significantly lower than the international age-standardized average, a trend that has been apparent for the last 30 years. The reason given for this was the high sociodemographic index of the country and that preventive actions are working [28]. These factors may help to explain why the development among the conscripts is favorable, and no deterioration has been observed in the study group, either. In Finland, youth inequality has been growing slightly [29]. There is also an association between inequality and oral health [30]. Together, these factors may also explain the concentration of the caries burden on an smaller proportion of the population. Furthermore, the findings of previous studies (Figure 2) show that caries burden is concentrated in an even smaller group of population.

The prevalence of the first signs of erosive tooth wear (BEWE 1–2) in subjects was high (38.1%). The number of those with erosive tooth wear requiring non-invasive (BEWE 0–8) and invasive (BEWE 9–18) therapy was also clearly increased compared to the 1966 birth cohort study [31]. A Finnish birth cohort study published in 2016 found that the prevalence of mild erosive tooth wear (BEWE sum score 1–2) in men aged 44–46 years was 24.6%, and the prevalence of more severe ETW was 52.7% [31]. By contrast, Bartlett et al. [32] found in a study of seven European countries that the rate of ETW (BEWE 1–2) among men and women aged 18–35 years was 53.8%. The differences between countries were large, and the Finnish citizens (n = 300) ranked best when considering a BEWE value of 2–3, which included 17.7% of those examined [32]. From this point of view, tooth wear among Finnish men will clearly be a growing problem in the future.

Based on the results, it can be concluded that third molars were not filled but were primarily removed. On the other hand, the results suggest also that the reason for third molar extraction was more often something other indication than the need for restorative treatment. These findings are in line with the Finnish current care guidelines [33].

The weakness of this study was the smaller size of the study population compared to the previous studies. There would have been no possibility of increasing the number of subjects because of the COVID-19 restrictions in the Defence Forces in July 2021. The conduct of the examinations could not be postponed either because the military service schedule would not have allowed that. However, the material is representative of male conscripts born 2000–2002. This can be justified because the strength of the research is that the conscripts who participated originate from all the parts of Finland evenly and were randomly selected. Inclusion criteria were only limited for age. The age cohort in the study population also exhibited differences in caries prevalence. These differences should be monitored in the future whether or not this phenomenon continues. The cross-sectional nature of the study prevents using the findings to determine the causes of the reduction in the caries burden. Nor can the outcome be generalized for other age groups or other genders. Further research is needed to determine the factors associated with the reduction of the caries burden.

The age distribution of the conscripts surveyed in the Ankkuriemi and Läärä studies [5,6] differs slightly between 2011 and this study; therefore, conclusions should be drawn with caution.

In general, epidemiological studies have not been performed on a Finnish population born at the turn of the 2000s. Estimates of the caries experience of young Finnish adults are based on values indirectly derived from many different registers. Therefore, making reliable comparisons of the caries experience of young Finnish adults to international results should be done with caution.

## 5. Conclusions

The oral health surveys conducted among conscripts in Finland from 1976 to 2011 show that that the caries burden has continued to decrease. However, the restorative treatment need resulting from caries has not disappeared from the population, and the burden is concentrated on an even smaller group of people. Moreover, common findings of the first signs of erosive tooth wear or of mild tooth wear in young men do not predict a reduction in the need for restorative treatment in the future.

## Figures and Tables

**Table 1:** Summary of previous cross sectional studies conducted on Finnish conscripts—1979–2011.

Data	Sample n	Ages	Gender	3rd Molars	Mean	SD	99% C.I. for Mean	DT > 0
1976 [5]	3344	17–29	M	Yes	4.34	5.03	4.25, 4.43	75.6%
1993 [6]	2850	19–20	M	Yes	1.98	3.08	1.92, 2.04	56.0%
2011 [7,8]	13564	19–21	M	No	1.40	2.50	1.38, 1.43	45.1%

**Table 2:** DMFT values with and without third molars.

		3rd Molars Included				3rd Molars Excluded			
Year of Birth		DT	MT	FT	DMFT	DT	MT	FT	DMFT
	n	Mean (SD)	Mean (SD)	Mean (SD)	Mean (SD)	Mean (SD)	Mean (SD)	Mean (SD)	Mean (SD)
2000	39	2.69 (3.00)	0.15 (0.67)	2.69 (3.26)	5.54 (4.78)	2.54 (2.93)	0.03 (0.16)	2.67 (3.20)	5.23 (4.77)
2001	159	1.49 (3.00)	0.30 (0.77)	2.37 (2.76)	4.14 (4.28)	1.34 (2.76)	0.09 (0.33)	2.36 (2.76)	3.80 (4.06)
2002	310	0.75 (1.59)	0.16 (0.64)	1.57 (2.20)	2.48 (3.21)	0.67 (1.52)	0.03 (0.19)	1.57 (2.20)	2.28 (3.05)
All	508	1.13 (2.31)	0.20 (0.67)	1.91 (2.51)	3.23 (3.83)	1.03 (2.17)	0.05 (0.24)	1.91 (2.50)	2.98 (3.66)
95% CI		(0.93, 1.33)	(0.14, 0.26)	(1.69, 2.13)	(2.90, 3.57)	(0.84, 1.21)	(0.03, 0.07)	(1.69, 2.12)	(2.66, 3.30)

**Table 3:** Distribution of DT (**a**) and DMFT (**b**) values with and without third molars.

**(a)**		**3rd Molars Included**		**3rd Molars Excluded**	
		**DT = 0**	**DT > 0**	**DT = 0**	**DT > 0**
**Year of Birth**	**n**	**% (n)**	**% (n)**	**% (n)**	**% (n)**
2000	39	35.9 (14)	64.1 (25)	35.9 (14)	64.1 (25)
2001	159	55.3 (88)	44.7 (71)	56.6 (90)	43.4 (69)
2002	310	71.3 (221)	28.7 (89)	73.2 (227)	26.8 (83)
All	508	63.6 (323)	36.4 (185)	65.2 (331)	34.8 (177)
**(b)**		**3rd Molars Included**		**3rd Molars Excluded**	
		**DMFT = 0**	**DMFT > 0**	**DMFT = 0**	**DMFT > 0**
**Year of birth**	**n**	**% (n)**	**% (n)**	**% (n)**	**% (n)**
2000	39	17.9 (7)	82.1 (32)	20.5 (8)	79.5 (31)
2001	159	19.5 (31)	80.5 (128)	22.0 (35)	78.0 (124)
2002	310	39.0 (121)	61.0 (189)	41.0 (127)	59.0 (183)
All	508	31.3 (159)	68.7 (349)	33.5 (170)	66.5 (338)

**Table 4:** Distribution of BEWE score values.

	BEWE Score			
Year of Birth	0	1–2	3–8	9–13
	% (n)	% (n)	% (n)	% (n)
2000	40.5 (15)	27.0 (10)	27.0 (10)	5.4 (2)
2001	42.4 (64)	32.5 (49)	25.2 (38)	0.0 (0)
2002	36.3 (111)	42.2 (188)	20.3 (62)	1.3 (4)
All	38.5 (190)	38.1 (188)	22.3 (110)	1.2 (6)

## Data Availability

Restrictions apply to the availability of this data, which were obtained from the Finnish Defence Forces. Data are available from the corresponding author with the separate permission of the Finnish Defence Forces.
