# Correction: Patinen et al. Caries Experience and Erosive Tooth Wear in Finnish Men Conscripts 2021: A Cross-Sectional Study. *Dent. J.* 2022, *10*, 122

**DOI:** 10.3390/dj10110197

**Published:** 2022-10-24

**Authors:** Pertti Patinen, Tarja Tanner, Mika Huttunen, Annakaisa Muhonen, Sari Räsänen, Pernelle Moilanen, Jari Päkkilä, Vuokko Anttonen, Antti Kämppi

**Affiliations:** 1 Research Unit of Oral Health Sciences, University of Oulu, Aapistie 5, 90220 Oulu, Finland; 2 Finnish Defence Forces, Centre for Military Medicine, Tykkikentäntie 1, 11311 Riihimäki, Finland; 3 Research Unit of Mathematical Sciences, University of Oulu, Pentti Kaiteran katu 1, 90014 Oulu, Finland; 4 Department of Oral and Maxillofacial Diseases, University of Helsinki, Haartmaninkatu 1, 00014 Helsinki, Finland

## Text Correction

There was an error in the original publication [1]. Some written errors need to be corrected.

A correction has been made to the following sections:

Abstract:

“Most (76.6%) had BEWE (basic erosive wear examination) of 0–2.”

Results, Paragraph 3:

“Large proportion of conscripts examined, 38.1%, had the first signs of erosive tooth wear (BEWE 1–2) visible in their dentition.”

Discussion, Paragraph 3:

“On the other hand, the results suggest also that the reason for third molar extraction was more often something other indication than the need for restorative treatment.”

## Table Legend

In the original publication, there was a mistake in the legend for Table 4. The correct legend appears below.

**Table 4.** Distribution of BEWE score values.

## Error in Table

In the original publication, there was a mistake in Table 2 as published. The data are misplaced. The corrected Table 2 appears below.

The authors state that the scientific conclusions are unaffected. This correction was approved by the Academic Editor. The original publication has also been updated.

## Figures and Tables

**Table 2:** DMFT values with and without third molars.

		3rd Molars Included				3rd Molars Excluded			
Year of Birth		DT	MT	FT	DMFT	DT	MT	FT	DMFT
	n	Mean (SD)	Mean (SD)	Mean (SD)	Mean (SD)	Mean (SD)	Mean (SD)	Mean (SD)	Mean (SD)
2000	39	2.69 (3.00)	0.15 (0.67)	2.69 (3.26)	5.54 (4.78)	2.54 (2.93)	0.03 (0.16)	2.67 (3.20)	5.23 (4.77)
2001	159	1.49 (3.00)	0.30 (0.77)	2.37 (2.76)	4.14 (4.28)	1.34 (2.76)	0.09 (0.33)	2.36 (2.76)	3.80 (4.06)
2002	310	0.75 (1.59)	0.16 (0.64)	1.57 (2.20)	2.48 (3.21)	0.67 (1.52)	0.03 (0.19)	1.57 (2.20)	2.28 (3.05)
All	508	1.13 (2.31)	0.20 (0.67)	1.91 (2.51)	3.23 (3.83)	1.03 (2.17)	0.05 (0.24)	1.91 (2.50)	2.98 (3.66)
95% CI		(0.93, 1.33)	(0.14, 0.26)	(1.69, 2.13)	(2.90, 3.57)	(0.84, 1.21)	(0.03, 0.07)	(1.69, 2.12)	(2.66, 3.30)

## References

[1] Patinen P., Tanner T., Huttunen M., Muhonen A., Räsänen S., Moilanen P., Päkkilä J., Anttonen V., Kämppi A. (2022). Caries Experience and Erosive Tooth Wear in Finnish Men Conscripts 2021: A Cross-Sectional Study. Dent. J..

